# Assessment of the Efficiency of Consolidation Treatment through Injections of Expanding Resins by Geotechnical Tests and 3D Electrical Resistivity Tomography

**DOI:** 10.1155/2015/237930

**Published:** 2015-06-17

**Authors:** T. Apuani, G. P. Giani, M. d'Attoli, F. Fischanger, G. Morelli, G. Ranieri, G. Santarato

**Affiliations:** ^1^Department of Earth Sciences “Ardito Desio”, University of Milan, Via Mangiagalli 34, 20133 Milan, Italy; ^2^Geosec S.r.l., Via G. di Vittorio 41/b, 43044 Collecchio, Italy; ^3^Geostudi Astier S.r.l., Via A. Nicolodi 48, 57121 Livorno, Italy; ^4^Department of Civil Engineering, Environmental Engineering and Architecture, Via Marengo 2, 09123 Cagliari, Italy; ^5^Department of Physic and Earth Sciences, Via Saragat 1, 44122 Ferrara, Italy

## Abstract

The design and execution of consolidation treatment of settled foundations by means of injection of polyurethane expanding resins require a proper investigation of the state of the foundation soil, in order to better identify anomalies responsible for the instability. To monitor the injection process, a procedure has been developed, which involves, in combination with traditional geotechnical tests, the application of a noninvasive, geophysical technique based on the electrical resistivity, which is strongly sensitive to presence of water or voids. Three-dimensional electrical resistivity tomography is a useful tool to produce effective 3D images of the foundation soils before, during, and after the injections. The achieved information allows designing the consolidation scheme and monitoring its effects on the treated volumes in real time. To better understand the complex processes induced by the treatment and to learn how variations of resistivity accompany increase of stiffness, an experiment was carried out in a full-scale test site. Injections of polyurethane expanding resin were performed as in real worksite conditions. Results confirm that the experimented approach by means of 3D resistivity imaging allows a reliable procedure of consolidation, and geotechnical tests demonstrate the increase of mechanical stiffness.

## 1. Introduction

The application of soil consolidation techniques using resins has greatly increased over recent years, especially following the development of new materials and methods specifically studied for this field of application. The purpose of the resin-based consolidation techniques is to remove the causes of settlements of the foundations by injecting an expanding polymer material into the ground.

The first procedures implemented date back to the second half of the 1950s in the United States and to the 1990s in Europe. Now they are available on the market for a variety of applications. Among the most popular procedures employed there are consolidation techniques, involving the free diffusion of resin in the ground, and mixed techniques, combining the resin injected with a load-bearing structural element connected with the existing foundation for the transfer of the load to a given depth. The interest aroused by this procedure lies in its high efficiency, as shown by the following facts: (i) it provides an effective solution, even in the long term; (ii) it is minimally invasive as it allows rapidity and flexibility of the treatment and the ability to work in narrow spaces using streamlined and compact instruments; (iii) it is environmentally compatible.

Settlements of the ground subjected to the foundation load are often due to periods of drought alternating with heavy rainfalls, sewer leaks, heavy vehicle traffic, excavations close to the building, and growth of tree roots and variation of applied loads. The interaction between water and foundation soil is undoubtedly one of the most important mechanisms and a critical factor for all types of ground. Clayey soils, in fact, have the capacity to absorb large quantities of water, which leads to a considerable increase in volume. On the other hand, loss of water manifests itself in a decrease in the volume of the ground. If periods of heavy rain alternate with periods of drought, the volume of the ground is cyclically subjected to swelling and shrinkage [1], which leads to the creation of voids, hence to the risk of differential displacement. Granular soils, in contrast, exhibit greater permeability and the water circulates freely, possibly transporting fine particles. This circulation, if sufficiently intense, can progressively lead to the creation of voids and cavities, which often contribute to the phenomenon of foundation settlements.

Many factors affect the process of soil consolidation by injections of expanding resins: on one side the geotechnical and hydrogeological context and on the other the specific resin-based consolidation technique. Although the use of resin-based consolidation techniques is more and more growing, the full comprehension of the process involved is not completely achieved yet. Some authors have presented case studies in different geological contexts, using specific resin and injection techniques [2, 3]. Some studies are devoted to explore the variation of geotechnical, hydrogeological, and geophysical properties of soils, during and after the treatment, by a back analysis approach on dedicated test sites [4, 5] or real application cases [6, 7]. Only few authors have applied a theoretical approach to predict the treatment efficiency [8]. It results that the topic needs to be scientifically investigated further.

Due to the complexity of the system (soil/resin), it is unavoidable that each study is dependent on the geotechnical context as well as on the product and procedure used. To shed light on the observed correlation between geotechnical and geoelectrical response variations due to resin injection that allow to use time-lapse resistivity surveys to suitably monitor the injection process, a full-scale field test was planned. Here injections of two-component polyurethane expanding resin were executed, following the procedure already described by Santarato et al. ([6]; see also [9]). Detailed geotechnical tests were performed before, during, and after the injections, and repeated three-dimensional electrical resistivity tomography (3D-ERT) was performed as well.

The test site was purposely selected to analyse what occurs in recovery of foundation settlements by means of resin injection in cohesive soils, which is a very frequent although specific occurrence in the consolidation practice.

## 2. The Test Site: Geological Outline

A full-scale test site was set up, where resin injections were carried out in compliance with the usual procedure followed in real cases of settlements of building foundations.

**Figure 1:**
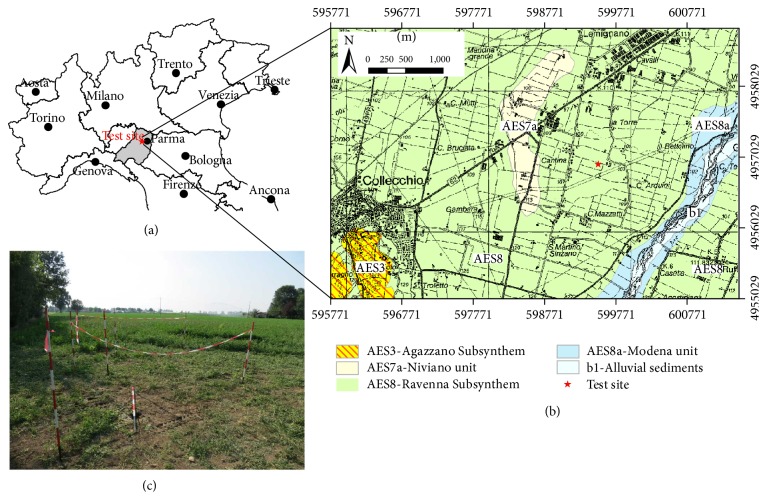
(a) Test site location in Italy. (b) Geological map of the area (ISPRA, Sheet 199: coordinates are in meters, ED50). (c) View of the test site.

The test site is an agricultural field, presently uncultivated, located south-west of Parma, in the Municipality of Collecchio (Figures 1(a) and 1(c)). From the geological point of view, it lies in the foredeep of the Apennine chain, which rises to the immediate south of the site location and develops in a NW-SE direction, bordering the southern side of the Po Valley plain.

The geology of the area (Figure 1(b)) is characterised by a marked accumulation of quaternary alluvial sediments, which lie in simple discordance on the substratum of late Pliocene and early Pleistocene marine sediments. The depth of these sediments narrows down noticeably moving towards the SW and reaches its minimum depth near Collecchio town. In the site location the shallowest stratigraphic unit, belonging to the upper emiliano-romagnolo Synthem (AES), is the Ravenna Subsynthem (AES8) [10] with a maximum thickness of about 20 m. The AES8 unit is constituted by gravel, sand, and layered silts: alluvial fan and intravalley terraced deposits or deposits of the secondary hydrographic network, covered by clay silts, which represent palaeosoils at varying degrees of evolution; local lenses of gravel and sand reach, on average, a thickness of 3 metres.

The hydrogeological arrangement of the area is outlined in three complexes: the gravelly-sandy upper complex (A), the sandy-clay complex (B), and the basal clay complex (C). The coarse horizon of the AES8 unit constitutes the first main aquifer (A), and the water level in the site location was estimated to lie at about 10 m below ground level (b.g.l.) (regional monitoring network).

## 3. Hints about the ERT Geophysical Method

Electrical resistivity of loose sediments depends mainly on their water content, besides their texture and mineral composition, as it is well known (among many others: [11–14]), so that electrical resistivity is the best-suited geophysical parameter that can be used for characterisation of water content status and of its changes in space and time.

The three-dimensional (3D) electrical resistivity tomography (ERT) is a geophysical imaging method, which allows determining the distribution of electrical resistivity in the volumes of the investigated subsurface. Electrical potentials produced by a continuous current input into the soil are measured, using two couples of electrodes driven into the soil, the first one to feed a continuous electric current and the second one to measure the potential drop, whose value is determined by the distribution of resistivity in the investigated subsoil (and by the reciprocal positions of the electrodes. obviously). The generalized use of the modern automatic multielectrode georesistivity meters allows to manage many (tens and even hundreds) electrodes, laid out in the surveyed area, so that hundreds and event thousands of measurements can be harvested in very short times [15]. In order to obtain the distribution of resistivity in the subsurface, inversion strategies are used. These involve first the discretization of the investigated subsurface into (generally hexahedral) blocks and the numerical solution, through finite element techniques, of the Laplace's differential equation, which rules the phenomenon:

(1) $$\begin{array}{c} \nabla^{2}V=0, \end{array}$$

where *V* is the electrical potential at a distance *r* from a point source of steady electrical current *I*. The whole dataset of measurements is then simulated and compared to data, by means of an optimization procedure according to a recursive least squares approach, so that the resistivity distribution in the investigated subsurface is estimated. For the whole cycle of inversions a customised version of ERTLAB was used, which uses a robust approach to least squares minimisation, as described by Morelli and LaBrecque [16] and the a priori *χ* ^2^ statistic as a misfit criterion: therefore convergence is reached when *χ* ^2^ approaches the number of data. Any lithological variations, states of soil saturation, cavities, buried structures, or other anomalous bodies, which are characterised by different resistivity values or ranges, are therefore imaged and can be highlighted, in compliance with the specific investigation depth and the resolving power of this method.

Based on these considerations, ERT, as an indirect noninvasive technique, is ideal for monitoring the injection treatment while it is being carried out. The mechanism by means of which the polyurethane resins (which have high electrical resistivity) are distributed in the soil normally follows a dendritic pattern (see Figure 7). Therefore the filaments of resin do not have any significant impact on the flow of electrical current in the subsurface; as a consequence the resistivity variations, observed along time while injections are carried out, are to be attributed to the effect of the removal of the interstitial water and/or the filling of voids, as a result of the compaction triggered by the expansion of the resin.

## 4. Methodology: Design of Injection and Investigations

**Figure 2:**
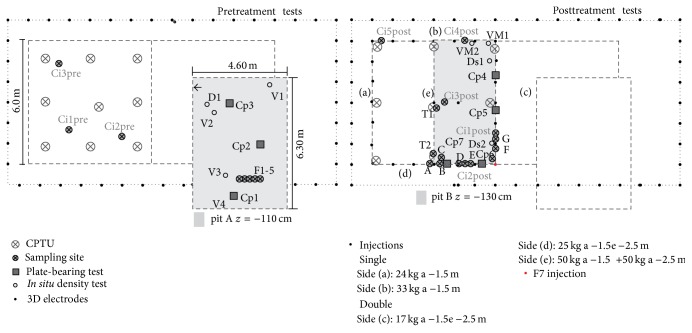
Map of the test field showing location and specifications of samplings and geotechnical-geoelectrical tests. The test or sampling identity codes concern: V = balloon method density test; D = sand cone method density test; VM = posttreatment balloon method density test; Ds = posttreatment sand cone method density test; Cp = plate-bearing test; F = sampling with perforating punch: CPTU = cone penetration test.

In the test site, two adjacent square plots, each measuring 6 m per side, were selected, covering a total surface area of 6 × 12 m² (Figure 2). Consolidation treatments were carried out in the first plot while no treatments were executed in the second one, kept undisturbed for comparison purposes.

In order to assess the modifications brought about by injecting resin into the ground, both areas were subjected to geotechnical and geophysical testing aimed at identifying the soil nature and the relative strength and deformability parameters. The geotechnical* in situ* tests have included cone penetration tests CPTU and plate-bearing tests, which were carried out before and after the injections. In addition geotechnical laboratory tests were performed on samples of “undisturbed” soil (direct shear test and unconfined compressive test) and on “resin-treated” soil (unconfined compressive test). Likewise, in compliance with worksite practices, 3D ERT measurements were carried out in order to acquire, even in volumes of soil not directly controlled by geotechnical testing, the information that is normally necessary for the treatment design of the consolidation procedure and its monitoring in real time. The strength and deformability characteristics of soil, before and after the procedure, deduced by the geotechnical surveys were thus compared with the geophysical imaging results.

The consolidation treatment involved executing 39 injections, carried out mainly at a depth of 150 cm below ground level (b.g.l.), along the perimeter of the 6 × 6 m² area involved in the treatment process, with a distance between centres of 1 metre. A number of repeat injections in the same point were carried out at a depth of 250 cm from ground level. The resin used, called “Eco-Maxima,” has considerable compressive strength in addition to good tensile and buckling strength. Uniaxial compressive tests conducted by the University of Parma and at the facilities of the R&S laboratory of Parma on resin samples of different densities (from 40 to 200 kN/m^3^) produce strength values between 133 and 2420 kN/m^2^.

Geotechnical surveys were carried out in three consecutive stages.

On the day dedicated to consolidation treatment, CPTU static penetration tests were carried out before, during, and after the injections, for a total number of 27 tests (9 preliminary, 9 intermediate, and 9 posttreatment tests). Furthermore, cores of treated and nontreated soils were sampled (Figure 2). The whole consolidation phase was monitored using 39 repeated 3D ERT surveys corresponding to the intermediates carried out following each injection of resin. An array composed of 48 electrodes, spaced 1 metre apart, was laid out over an 8 m × 16 m rectangle surrounding the two disturbed and undisturbed plots in the test site. This layout guarantees an investigation depth of about 3 m, which includes the whole volume of soil affected by resin injection, as purposely designed to simulate realistic working conditions. In such specific this depth range and contenxt [6], the reduction of resolving power is negligible, if compared with conventional ERT data acquisition by means of a regularly spaced electrode grid.

The comparative analysis of all the surveys made it possible to identify the lithostratigraphic properties of the site and indirectly to obtain specific soil parameters by preserving the soil from invasive excavation. It is obvious that both core sampling and penetration tests led to some modifications, although minimum, to the mechanical characteristics of the ground nearby the investigation points. This was duly taken into consideration when selecting the areas for executing subsequent tests.

In a second stage, excavation and soil removal were carried out down to a depth of 110 cm b.g.l. (pit A in Figure 2) in the area adjacent to the ground treated with resins. The aim was to obtain exposed stratigraphic sections and, above all, a level surface on which to carry out geotechnical density tests and plate-bearing tests in addition to pretreatment characterisation.

A third investigative stage consisted in excavating a portion of soil down to a depth of 130 cm b.g.l. (pit B in Figure 2), thus intervening on the volumes of soil affected by the injections. It was therefore possible to directly observe the distribution of the resins in the soil, and let exposed a level surface on which to sample and to carry out the same* in situ* tests before and after treatment.

## 5. Geotechnical and Geophysical Pretreatment Characterisation of the Test Site

The information about the nature of the subsurface, the stratigraphy, and the grain size distribution of involved soils derives from the joint analysis of (i) surveys conducted on the pit A, sampling and geotechnical identification tests; (ii) CPTU penetration tests; (iii) pretreatment 3D ERT survey; (iv) geotechnical laboratory and* in situ* tests aimed to obtain soil strength and deformability.

**Figure 3:**
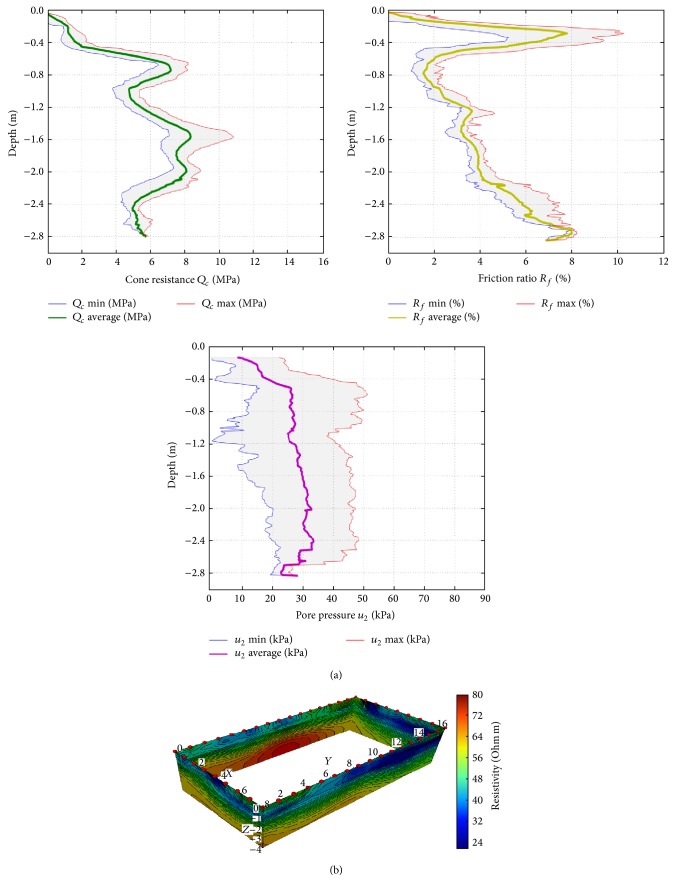
(a) Cumulative results of CPTU geotechnical tests performed before injections: cone penetration resistance *Q*_*c*_, friction ratio *R*_*f*_, and pore pressure *u*_2_; (b) fence diagram of the 3D ERT resistivity model before injections.

(i) Excavation of pit A brought to the light continuous and uniform stratigraphic characteristics all along the perimeter. Two horizons can be discerned: the first at a depth of approximately 40–50 cm consists of brown clay and silt with a uniform particle size with sparse millimetric clasts. The second horizon extends to the bottom of the pit and is made up of brownish-yellow plastic and very firm silty clays. Root structures with diameters measuring in the region of centimetres are found down to 70 cm b.g.l. The results of the geotechnical tests refer to samples taken at a depth around 150 cm. The investigated soil can be defined as* silt with clay*,* slightly sandy*, or rather* CL* according to the USCS (Unified Soil Classification System, adopted by [17]), organic content O = 5–10%, nonuniform (coefficient of uniformity Cu = 9–18), medium plasticity (liquid limit WL = 40–53% and plasticity index PI = 22–26%), with water content W = 13–23% in natural conditions, firm or locally very firm. Density tests carried out* in situ* using the sand cone method [18] and rubber balloon method [19] or in laboratory using the drive cylinder method [20] provide weight values of natural volume *γ*_*o*_ = 19.3 ± 0.7 kN/m^3^, dry weight volume *γ*_*d*_ = 16.1 ± 0.9 kN/m^3^, a calculated porosity *n* = 34 ± 5%, the measured absolute specific weight being *γ*_*s*_ = 25.3 ± 0.5 kN/m^3^ (mean values ± standard deviation).

(ii) The nine CPTU tests performed preliminary to the injections reached a maximum depth of approximately 280 cm b.g.l. Plots in Figure 3(a) display the averaged profiles and data dispersions (grey areas) for cone resistance (*Q*_*c*_), friction ratio (*R*_*f*_), and pore pressure (*u*_2_) parameters. Both cone resistance and friction ratio values show a good consistency at the same depth for different CPTU tests. Only variations of the order of 20% are observed, which are due to slight lateral inhomogeneity of the layers. The maxima variation in *Q*_*c*_ is recorded at a depth of 160 cm b.g.l., while that in *R*_*f*_ is located at about 30 cm b.g.l. where tree roots were locally revealed by direct surveys conducted on pit A. The pore pressure parameter shows wider variations, probably related to local conditions, and to the higher sensitivity of it to different injection procedures. For this reason, a strict comparison of the absolute values of *u*_2_ among tests is not achievable; anyway, in general, all tests show a weak gradual increase of the pore pressure with depth.

(iii) Figure 3(b) shows, as a fence diagram, the distribution of resistivity below the layout of electrodes as obtained by 3D inversion of the data acquired before injections. The information provided by the 3D ERT is coherent with the evidence provided by the excavation and by the CPTU tests performed before the injection sequence.

The following electrostratigraphic layers, consistent with the penetrometric test results and with the usual range for loose lithologies in alluvial plains [21], can be seen:

- a shallowest layer, down to 40–50 cm: aerated soil, disturbed by farming activities, defined by geotechnical investigations as* brown silt with clay*, with resistivity in the range of 50–60 Ohm·m characterized by the lowest values of cone penetration resistance, a depth increasing side friction (*F*_*s*_), and the highest friction ratio (*R*_*f*_);
- an intermediate layer, from 40 to 150 cm:* plastic silty clays*, with resistivity values in the range 20–40 Ohm·m, *Q*_*c*_ values ranging from 3 to 7 MPa, a friction ratio (*R*_*f*_) slightly increasing in depth;
- a more resistive layer, below about 2.4 m:* silt with clay*,* slightly sandy*, characterized by an increase in resistivity up to values around 70 Ohm·m, average *Q*_*c*_ values of 8 MPa, with higher values of both resistivity (up to 100 Ohm·m) and cone resistance (up to 10 MPa) related to the presence of larger particle size soils (partially saturated sand and gravel).

Further investigations and discussion will be focused on the intermediate layer.

Further geotechnical laboratory and* in situ* tests were performed on samples referring to the intermediate layer between −100 and −150 cm, corresponding to the more conductive lithology (25–30 Ohm·m), associated with the more abundant presence of fine material. Soil shear strength parameters, obtained by direct shear tests [22] on undisturbed samples furnished, respectively, are peak cohesion *c*′ = 42–74 kPa and peak friction angle *ϕ*′ = 17–21°. Unconfined compressive tests, executed before and after treatment, were considered a more suitable tool for evaluating the effectiveness of the treatment, in a similar way to that suggested in the assessment for checking inertization treatments [23] and considering the engineering problems for which the treatments are executed. The investigated soil has a uniaxial compressive strength *σ*_*c*_ = 208 kPa (mean value of 6 samples) with *σ*_*c*_ = 159–464 kPa and deformation modulus calculated at 15% of *σ*_*c*_ *E*_15%_ = 14.0–31.6 MPa. The high extreme values of strength and stiffness are ascribable to samples with a certain amount of disturbance caused by the sampling process.

The elasticity values obtained on the sampling scale are consistent with the behaviour detected by means of the plate-bearing tests executed at the bottom of the pit B (*z* = −130 cm) following the CNR Mod T0116/A [24] standards, using a 30 cm diameter plate, executing two load cycles with maximum load 0.35 MPa spaced out by a rapid unload phase. The* in situ* tests provide the following compressibility moduli: *M*_*E*_ = 13.9–22.4 MPa and *M*_*E*_′ = 26.6–31.1 MPa calculated on the first and second load curve, respectively.

## 6. 3D ERT Monitoring of the Injection Process

The whole consolidation phase was monitored using 3D ERT surveys together with the acquisition of 39 sets of ERT measurements corresponding to the intermediates carried out following each injection of resin.

In a previous paper [6] the large-scale variation of the subsoil resistivity was described, during a resins injection treatment in real worksite conditions. For the test site described here, we will show selected ERT monitoring images, focused on a smaller scale, in order to infer, by means of a more detailed view, the dynamics of subsoil resistivity changes that follow the resins injection process and possibly to connect these features with evidences coming from geotechnical data. We will therefore describe the changes in resistivity values and distribution of a selected portion of the test site subject to a sequence of adjacent injections, monitored by consecutive ERT intermediate readings. This portion is representative of all other sets of injections and ERT measurements that were acquired on the site.

**Figure 4:**
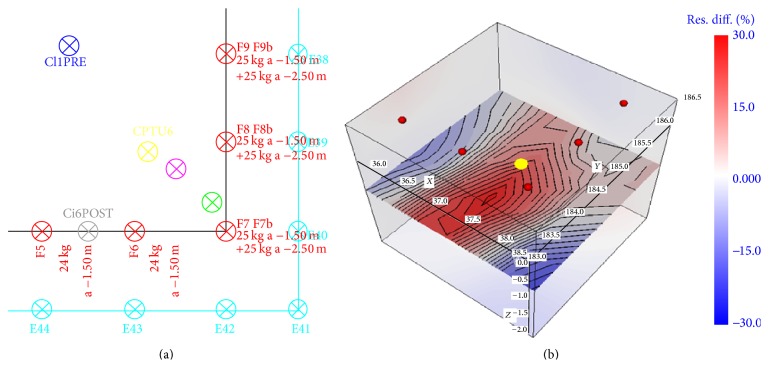
(a) Detail of the site area being analyzed for discussion of ERT time-lapse results (cyan: electrodes; red: injection points). (b) Detail of the resistivity changes at time T1 after injection F7, in the neighborhood of the injection point F7 (yellow dot). Horizontal slice at *z* = −1.5 m.

We focus on the ERT results and images within a time frame that covers a sequence of 3 injections—referred below as F6, F7, and F8 spatially adjacent at 1 m from each other (Figure 4(a))—selected on the basis of the following criteria:

1. these are the very first injections that were performed on the site, in order to operate in undisturbed background conditions to get a reliable reference for the resistivity changes;
2. the injected volume of soil is located close to one corner of the electrodes arrangement, within the best sensitivity portion of the investigated subsurface [6]. The array of electrodes is 1 m apart from the injections;
3. the three injections were performed at the same depth, −1.50 m b.g.l., using a similar amount of resins (24-25 Kg), with the injection sequence T1-F7, T2-F8, and T3-F6;
4. injections and subsequent ERT measurements were all made in the same day, within few hours, with the same spread of electrodes, the same cables arrangement, and the same instrument: this reduces the possibility that “external” factors can affect the resistivity data processing.

All 3D ERT measurements were acquired by using a 10-channel IRIS Syscal Pro georesistivity-meter and selecting the pole-dipole array, as it is the most suited in terms of resolving power for this specific acquisition of a 3D information [6]; asymmetrical response of this array was compensated by acquiring both “direct” and “reverse” sequences of measurements. All possible combinations of receiving dipoles with lengths 1, 2, 3, 4, and 5 meters were acquired for each transmitting electrode of the spread. A whole number of about 4600 measurements were acquired for each intermediate and, before inversion, submitted to data quality control and preprocessing steps, in order to remove low signal to noise data coming from the most unfavorable arrangements of electrodes. Inversion was then performed by using a finite element mesh of 0.5 meters size in *x*, *y*, and *z* directions, that is, half of the spacing between contiguous electrodes. In all inversions, carried out in compliance with the smoothness-constrained inversion approach [25], we used as starting model a homogeneous model of 55 Ohm·m with an estimated standard deviation error for the datasets equal to 2%. The inversions converged after 6 iterations, using as a misfit criterion the a priori *χ* ^2^ statistic, in this case equal to about 3900, the number of measurements retained after rejecting low-quality data, as specified above.

The Figure 4(b) shows a horizontal slice and the percent variations in resistivity, occurred in the neighborhood of an injection point (yellow), at T1 (after injection inF7) with respect to T0. An increase of resistivity is observed in the area of soil close to the injection point, while areas with decrease in resistivity are present when moving away from the injection point.

**Figure 5:**
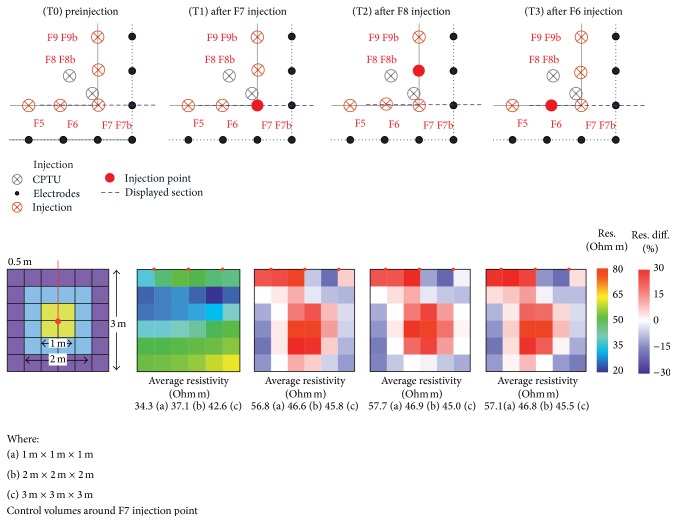
Variations of resistivity values at different intermediate times T1, T2, and T3, with respect to their preinjection T0 values in the 50 × 50 cm cells for the portion of the mesh around the F7 point. Average resistivities are calculated in increasing cubic volumes (1 m: average value (a), 2 m: average value (b), and 3 m: average value (c)). Injection characteristics: in F5, F6 24 kg at −1.50 m, in F7, F8 F9 25 kg at −1.5 m + 25 kg at −2.5 m.

In Figure 5 we show the variations of resistivity values at times T1, T2, and T3, with respect to their preinjection T0 values in the 50 × 50 cm cells at different intermediates for a portion of the mesh around the F7 point, under which the first injection was performed. The reported statistics refer to three, progressively increasing, volumes of soil in the neighborhood of point F7, with edges of 1 m, 2 m, and 3 m, respectively. Results are displayed on a vertical *xz* plane passing through the F7 injection point. A similar behavior can be observed in *yz* direction: there are no preferential directions of resins spread due to injection nozzles orientations since injection pipes are simply open at the base, so any preferential pathway of the resins is related only to the structural characteristics of the soil.

**Figure 6:**
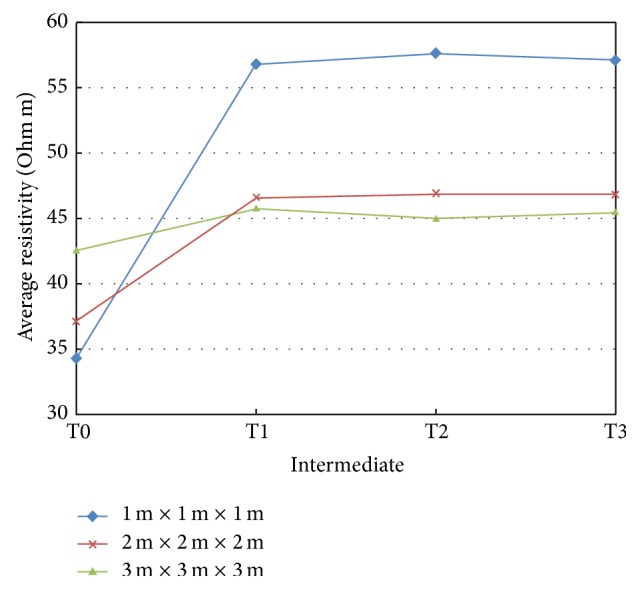
Average of resistivities at different intermediates for different volumes of soil around the control point.

Figure 6 illustrates the average resistivity at different intermediates (time) for volumes of 1 m, 2 m, and 3 m size in the neighborhood of the F7 injection point.

The above data and images allow to draw the following considerations.

**Figure 7:**
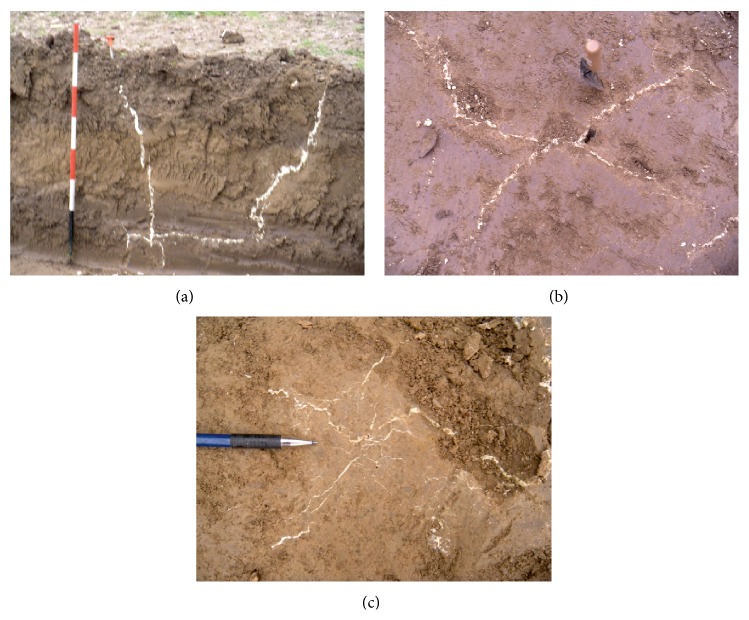
Effects of resin treatment: propagation of resins in clayey soils (a) main conduits along preexisting preferential weakness channels; (b) induced fracturing and spreading on a horizontal plane; (c) dendritic network at millimetric scale.

- Most of the increase in resistivity in the neighborhood of the “control point” occurs after the first injection.
- A decrease of resistivity is observed in the outer shell of the displayed volume of soil: we suggest that this decrease can be related to water migrated there, due to the effect of resin expansion. Water migration is consistent with evidences coming from geotechnical data (see Section 7), which show that the soil moisture conditions decrease significantly near the resin veins, as do the penetration resistance (pp) and the undrained cohesion (Cu) attest.
- The volume with the largest resistivity increase is contained in a radius of about 1 m around the injection point, with increases in resistivity sometimes close to 100%, for the given injected resin quantities and the site specific lithologies. This is consistent with visual information coming from the excavated volumes where it is possible to observe that, at the injection pressures, where no preexisting weakness channels exist, the channels of resin distribute themselves and invade a surrounding volume of radius approximately 1 m around the center of the injection. Moreover, these volumes correspond to those where the improvement of the mechanical properties was recorded by the geotechnical tests described below: this finding shows that increase in resistivity is due to the reduction in the void ratio and compaction of the soil.
- The T2 and T3 injections close to the “control point” seem only to act in stabilizing the T1 resistivity scenario, as if the nearest volume of soil did not undergo significant changes after the first injection.
- An increase of resistivity is also observed in the three surface blocks on the left side and a decrease in the remaining three on the right: this behavior may be attributed to resilience of resin (Figure 7), due to the lack of load, which in worksite conditions is given by the building.
- The average of the resistivity of progressively increasing volumes at different intermediates tends to a constant value: larger volumes are less affected by the effect of injections and probably the balancing effect of the resistivity decrease away from the injection point due to fluids displacements contributes to this result. Therefore, as it is shown that the consolidating action of the resin injection is confined to the immediate, approximately 1 m, neighbor of the injection point, spacing 1 m the injection points in worksite appears the proper choice.

## 7. Geotechnical Investigations after Treatment

The new excavation down to a depth of −130 cm b.g.l. (pit B in Figure 2) allowed to examine the volumes of soil affected by the injections and directly observe the created resin network; additionally it lets exposed a ground surface where it was possible to carry out the same* in situ* tests as it was done in pit A (without treatment).

It is clearly highlighted how the preexisting open discontinuities or channels constitute the preferential escape pathways for the distribution of the resins (Figure 7(a)). However, where none of these pathways is evident, induced fracturing systems can be spotted with characteristic directions. The configuration of the conduits, sometimes centimetres wide, filled by the hardened resin, complies with the directions of stress induced by the injections (Figure 7(b)). Furthermore a dendritic resin network is created which “strengthens” the adjoining soils (Figure 7(c)). Also the soil moisture conditions decrease significantly near these veins, as do the penetration resistance (pp) and the undrained cohesion (Cu) which, measured along the distance between centres of two adjacent injections (1 m), change, respectively, from pp *≅* 350 kPa with Cu *≅* 130 kPa to pp > 441 kPa with Cu > 240 kPa (instrumental end scale values). Visually, it is possible to observe that, at the injection pressures, where no preexisting weakness channels exist, the channels of resin distribute themselves and invade a surrounding volume of radius approximately 1 meter from the centre of the injection. Density tests, executed from the bottom of the pit nearby the injection axis, make it possible to obtain the weight volume at natural moisture *γ*_*o*_ = 19.2 ± 0.8 kN/m^3^ and the weight volume in dry conditions *γ*_*d*_ = 17.2 ± 1.1 kN/m^3^. Although the measurements depend on the entity of the resin “impregnating” the investigated volume, it is observed that the overall density of the treated soil remains substantially unchanged or does not in any case represent a significant parameter in assessing the efficiency of the treatment. This may be explained considering that the density of the resin, which has filled preexisting open fractures, is lower than that of water.

Even at the macroscale, samples subjected to laboratory geotechnical tests have different degrees of resin “impregnation”; resins may pass through the sample in veins or impregnate it in a uniform manner; samples subjected to tests are distinct depending on the percentage of resin present (<10%, 10–30%, and >30%).

**Figure 8:**
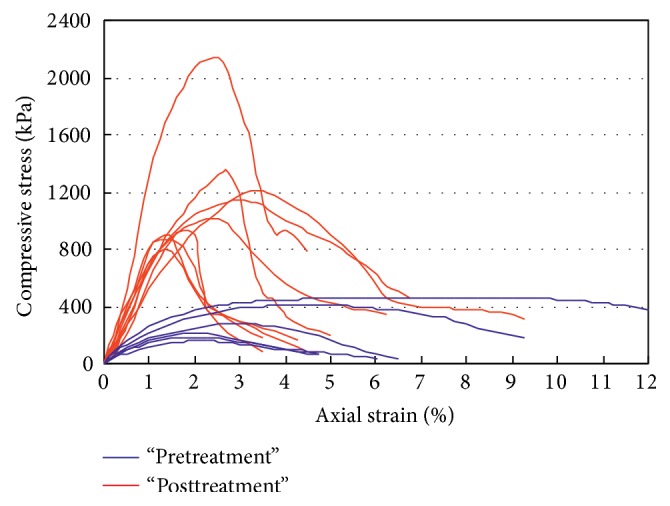
Stress-strain curves from unconfined compression tests refer to “pretreatment” samples and to “posttreatment” samples.

In Figure 8 the stress-strain behaviour revealed by means of unconfined compression tests on nontreated and treated samples is compared. The treated samples investigated have a compressive strength of *σ*_*c*_ = 1147 kPa (mean value taken from 9 samples) with *σ*_*c*_ = 788–2136 kPa and average deformation modulus *E*_15%_ = 76 MPa with *E*_15%_ = 49.2–128.2 MPa, which amounts to an increase of over 300% in the failure strength values and approximately 250% in the elastic moduli.

**Figure 9:**
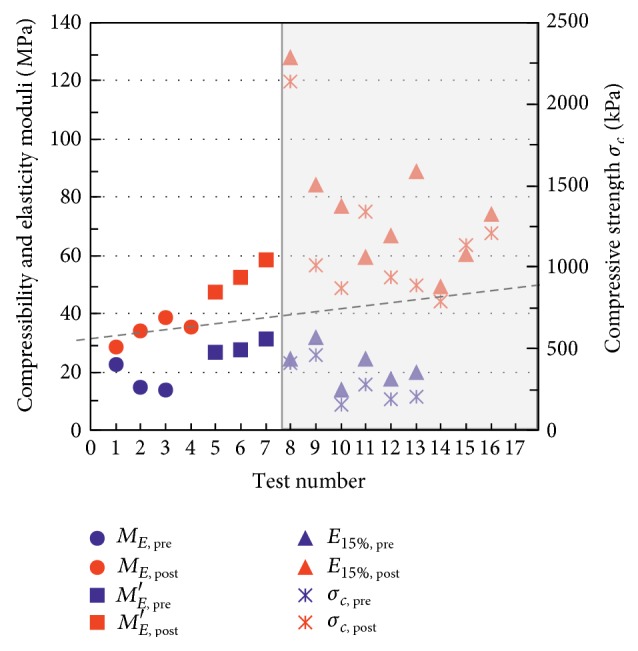
Comparison of the “pretreatment” and “posttreatment” geotechnical properties: compressibility moduli *M*_*E*_ and *M*_*E*_′ (from the first and second test cycle of plate-bearing test, resp.), elastic moduli (*E*_15%_) and unconfined compressive strength *σ*_*c*_.

Also comparing the compressibility values obtained through plate-bearing tests (Figure 9), executed at the bottom of the pit between two identical treatment injections, it is possible to appreciate a considerable increase in the bearing capacity of the soil with *M*_*E*_ = 28.5–38.6 MPa and *M*_*E*_′ = 47.6–58.7 MPa calculated on the first and second load curves, respectively. The dotted line in Figure 9 delimits the field of the typical values of “pretreatment” (below) and “posttreatment” (above) soil. The data consistently reveal a certain scale effect: the shaded area in Figure 9 groups the laboratory tests performed on samples measuring 5 cm in diameter, while the load plate has a diameter of 30 cm.

**Figure 10:**
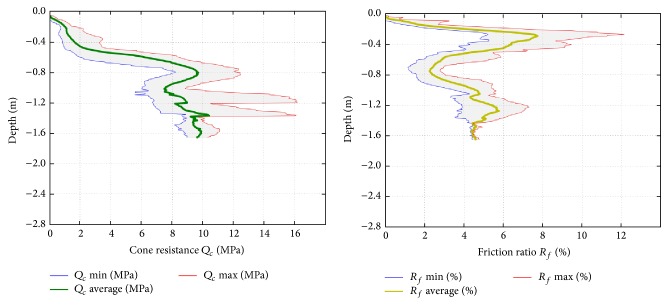
Cumulative results of CPTU geotechnical tests performed after injections: cone penetration resistance *Q*_*c*_, friction ratio *R*_*f*_.

The information provided by the CPTU tests carried out after the injection (Figure 10) is compatible with the evidence provided by the excavation and the geotechnical parameters at the sample scale. The CPTU tests show the achieved mechanical improvement around the treated area with the mean increase values of cone resistance (*Q*_*c*_), side friction (*F*_*s*_), and friction ratio (*R*_*f*_) indicated below:

(2) Qc,pre~7 MPa,Qc,post~9 MPa;Fs,pre~0.2 MPa,Fs,post~0.5 MPa;Rf,pre~2.5%,Rf,post~4.5%.

Focusing on the intermediate layer, high vales of *Q*_*c*_ (up to 16 MPa) are locally reached.

The CPTU results are also coherent with the information provided by the 3D ERT.

**Figure 11:**
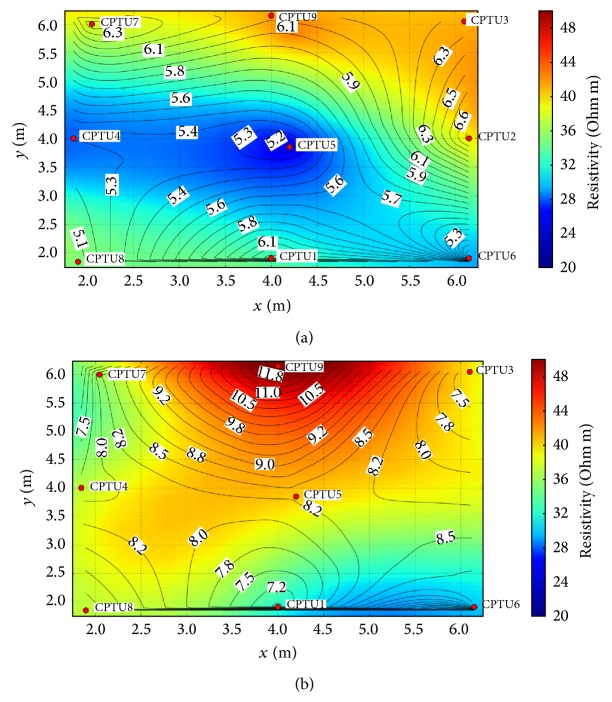
Comparison between pre- (a) and posttreatment (b) test results: horizontal section of the 3D resistivity model at 125 cm b.g.l. and *Q*_*c*_ contour lines (black lines) by interpolation at the same depth of all CPTU tests.

The efficiency of the treatment and the suitability of combing ETR monitoring and CPTU tests are confirmed by the comparison of pre- and posttreatment distribution maps of resistivity and cone resistance (*Q*_*c*_).  Figure 11 overlays the distribution of *Q*_*c*_ values before (a) and after (b) injections, on the horizontal section at the depth of 125 cm b.g.l., which corresponds to the shallowest minimum *Q*_*c*_ parameter, with the distribution of resistivity resulting from 3D resistivity model.

Considering the pretreatment results (Figure 11(a)) it can be noted that the relative inhomogeneity of the resistivity distribution ranges from values of 22 Ohm·m to 42 Ohm·m, and at the same depth the contour distribution of *Q*_*c*_ values quite accurately reproduces the resistivity distribution. This good correlation between these selected geotechnical and geophysical parameters is not surprising, because both parameters are strongly affected by grain size and compaction of loose sediments: their concordant variations correspond to variations of soil facies from prevailing clay content (smaller *Q*_*c*_ and resistivity) to prevailing silt content (greater *Q*_*c*_ and resistivity). Focusing on the posttreatments result map (Figure 11(b)), the two distributions are consistent and coherent. A general increase of both parameters is well evident: the increment of cone resistance is followed by an increase of resistivity. The distribution patters seem to be related to differences in the injection procedures (quantity and number of injections on the same vertical), in addition to the original pretreatment not perfectly uniform distribution.

## 8. Concluding Remarks

The experiments presented and discussed above were carried out through a multidisciplinary approach, which allowed recording the ways in which the process of soil consolidation by injecting expanding resins occurs and modifies mechanical properties of injected soil volumes. Volumes involved and effectiveness of the treatment were evaluated in terms of increasing mechanical resistance and stiffness, by means of standard geotechnical tests. Meanwhile, the injection sequence was monitored by successive 3D ERT measurement cycles, which allowed observing a remarkable variation of electrical resistivity, which in the present context is due to water movements. In particular, an increase of resistivity was recorded in treated volumes, accompanying the improvement of their mechanical properties, while a decrease of resistivity was observed in surrounding volumes, as the effect of the increase in their content of water, migrated from the injected volumes.

Direct inspection through observations made along the walls of the pit B has provided indications concerning the distribution and spread of the injected resins and contributed to support the 3D ERT as a noninvasive technique that is able to estimate the volumes involved in the process through compaction and migration of water held therein.

Both* in situ* and laboratory geotechnical tests have shown how the soils examined near the injected volumes significantly increase their bearing capacity, strength, and stiffness. It is the authors' opinion that the contribution provided by these injections is twofold: on one hand, it is linked to the reduction of the void ratio caused by the expansion of the resins and, on the other, by the presence of the resin itself, which compacts the treated soil in quite a uniform manner.

The results highlight the potential of integration between geotechnical analyses and geophysical surveys, in defining a geotechnical-geophysical procedure to be applied, both in the design and in the operative phases for assessing the consolidation in progress.

The discussion above allows confirming that the procedure followed in the test and already used in worksites [6, 7] carries to a possible “best practice” for the described procedure of planning and monitoring the consolidation of settled foundations by means of resin injections that can detailed as follows.

1. A 3D ERT survey is carried out in advance, in order to establish the initial conditions of the soil to be consolidated. The 3D representation of subsurface resistivity distribution, associated with the information deriving from the geotechnical survey (penetrometric or other tests) allows an initial general classification of the soils. The effectiveness of combining these survey techniques was recently confirmed by Dahlin et al. [26]. Moreover, the ERT provides details, regarding the type, continuity, and depth of the foundation, characterising any anomalies due to the presence of voids, water retention, or other distinctive lithological elements.
2. The preliminary geophysical information is integrated with the geotechnical and structural data available (crack pattern, geometry of the foundations, and loads) and contributes to the detailed design of the treatment procedure, in terms of location, number, depth of the injection holes, and quantities of resin to be injected.
3. In the course of the injection procedure, intermediate ERT surveys, carried out using the same electrode array, designed in phase 1, are conducted with the aim of investigating the status of the treatment of the soil volumes affected by the displacement and the variations in the fluid content in the subsurface, making it possible to redesign the treatment, practically in real time, on the basis of the emerging worksite evidence.
4. Just after completion of the procedure, a final 3D ERT verifies the new subsurface conditions after the consolidation procedure. In all steps, electrodes of the measuring layout must be kept fixed to ensure repeatability of results.
